# Improving Optical Temperature Sensing Performance of Er^3+^ Doped Y_2_O_3_ Microtubes via Co-doping and Controlling Excitation Power

**DOI:** 10.1038/s41598-017-00838-w

**Published:** 2017-04-07

**Authors:** Xiangfu Wang, Ye Wang, Jose Marques-Hueso, Xiaohong Yan

**Affiliations:** 1 College of Electronic Science and Engineering, Nanjing University of Posts and Telecommunications, Nanjing, 210023 People’s Republic of China; 2 Institute of Sensors, Signals and Systems, School of Engineering and Physical Sciences, Heriot-Watt University, Edinburgh, EH14 4AS UK; 3 School of Material Science and Engineering, Jiangsu University, Zhenjiang, 212013 People’s Republic of China; 4 College of Science, Nanjing University of Aeronautics and Astronautics, Nanjing, 211106 People’s Republic of China

## Abstract

This work presents a new method to effectively improve the optical temperature behavior of Er^3+^ doped Y_2_O_3_ microtubes by co-doping of Tm^3+^ or Ho^3+^ ion and controlling excitation power. The influence of Tm^3+^ or Ho^3+^ ion on optical temperature behavior of Y_2_O_3_:Er^3+^ microtubes is investigated by analyzing the temperature and excitation power dependent emission spectra, thermal quenching ratios, fluorescence intensity ratios, and sensitivity. It is found that the thermal quenching of Y_2_O_3_:Er^3+^ microtubes is inhibited by co-doping with Tm^3+^ or Ho^3+^ ion, moreover the maximum sensitivity value based on the thermal coupled ^4^S_3/2_/^2^H_11/2_ levels is enhanced greatly and shifts to the high temperature range, while the maximum sensitivity based on ^4^F_9/2(1)_/^4^F_9/2(2)_ levels shifts to the low temperature range and greatly increases. The sensitivity values are dependent on the excitation power, and reach two maximum values of 0.0529/K at 24 K and 0.0057/K at 457 K for the Y_2_O_3_:1%Er^3+^, 0.5%Ho^3+^ at 121 mW/mm^2^ excitation power, which makes optical temperature measurement in wide temperature range possible. The mechanism of changing the sensitivity upon different excitation densities is discussed.

## Introduction

Recently, optical temperature sensing behavior based on up-conversion luminescence of rare earth ion-doped phosphors have received much more attention since they can provide a non-contact temperature measurement in nanometer and submicron scale through probing the temperature-dependent fluorescence intensity ratio (*FIR*) of two adjacent thermally coupled energy levels^1–11^. The non-contact *FIR* technique is superior to the conventional temperature measurements, shows large spatial resolution and high accuracy of detection^12^. At present, trivalent rare earth ions, such as Er^3+^, Ho^3+^, Tm^3+^, Eu^3+^, and Pr^3+^, have been used as activators to study optical temperature sensing behaviors^13–18^. Among these ions, Er^3+^ is preferred for temperature sensing due to the large energy gap (about 800 cm^−1^) and small overlap of the two emission peaks from ^2^H_11/2_ and ^4^S_3/2_ levels^19–22^. So the optical thermometry based on up-conversion emission of Er^3+^ doped phosphors has been explored in different hosts by using infrared excitation sources^12–18^. These works reported that the optical temperature sensitivity of Er^3+^ doped phosphors was mainly dependent on host types, and lacked the investigation on the role of excitation powers and doping concentrations on the optical temperature behaviors.

However, Marciniak and Bednarkiewicz’s group observed that the highest sensitivity was reached 2.88%/K for LiYbP_4_O_12_:0.1%Er^3+^ nanocrystals upon pulsed excitation at average power below 25 mW/cm^2^, while the same material displayed lower ~0.5%/K sensitivity at higher 50–300 mW/cm^2^ excitation intensities^23^. Prasad’s group observed that the intensity ratio of green and red emissions were dependent on the excitation power density^24^. Li reported that the temperature sensing property of Er^3+^-Yb^3+^ co-doped NaGdTiO_4_ was dependent on the Er^3+^ concentration^25^. It was observed that the sensitivity values were strongly dependent on the excitation powers and excitation modes^2, 26^. Thus, it is interesting to systematically study the concentration and excitation powers dependent optical temperature behaviors in different materials.

Phosphors with high thermal stability are excellent candidate materials to be used for optical temperature sensing. Compared with the fluorides, Y_2_O_3_ has the advantages of a high melting point, wide bandgap, high solubility between Y^3+^ and Er^3+^, and good transparency in ultraviolet and infrared range^27^. Based on the green emissions from thermally coupled energy levels of ^2^H_11/2_ and ^4^S_3/2_, the optical temperature sensing was studied in Er^3+^-Yb^3+^ co-doped Y_2_O_3_ bulks and nano-materials^27–32^. However, the aforementioned works have not dealt with excitation powers dependent optical temperature sensitivity, and lack to explore how to improve the optical temperature behaviors. The optical temperature property of Er^3+^ doped phosphors was determined by the population of the thermally coupled energy levels of ^2^H_11/2_ and ^4^S_3/2_^12^. If the emission intensity ratios from the ^2^H_11/2_ and ^4^S_3/2_ is adjusted, the temperature sensitivity will change greatly. As reported that the energy gaps between ^3^F_4_ and ^3^H_6_ of Tm^3+^ ion, as well as between ^5^I_7_ and ^5^I_8_ of Ho^3+^ ion, were equal to the energy gap between ^2^H_11/2_ and ^4^F_9/2_ of Er^3+^ ion^19^. Thus, the population of ^2^H_11/2_ and ^4^S_3/2_ levels of Er^3+^ can be adjusted through the cross relaxation energy transfer between Er^3+^ and Tm^3+^ (or Ho^3+^). In this case the optical temperature sensitivity will be easy controlled through controlling the concentration of the Tm^3+^ or Ho^3+^ ion. Considering the structure of energy levels of Tm^3+^ and Ho^3+^, in this work, we propose a method to improve the optical temperature behavior of Er^3+^ doped Y_2_O_3_ through adjusting the green emission ratios with the cross-relaxation energy transfer between Er^3+^ and Tm^3+^ (or Ho^3+^). The Er^3+^ doped, Er^3+^-Tm^3+^ co-doped, and Er^3+^-Ho^3+^ co-doped Y_2_O_3_ microtubes are synthesized, and their optical temperature behaviors are studied by controlling the excitation power of the laser and the concentration of Tm^3+^ or Ho^3+^ ions. It is observed that the optical temperature sensitivity of Er^3+^-doped Y_2_O_3_ microtubes is significantly improved compared to the optical temperature sensitivity of Er^3+^-Yb^3+^ co-doped Y_2_O_3_ bulks and nano-materials.

## Results

**Figure 1:**
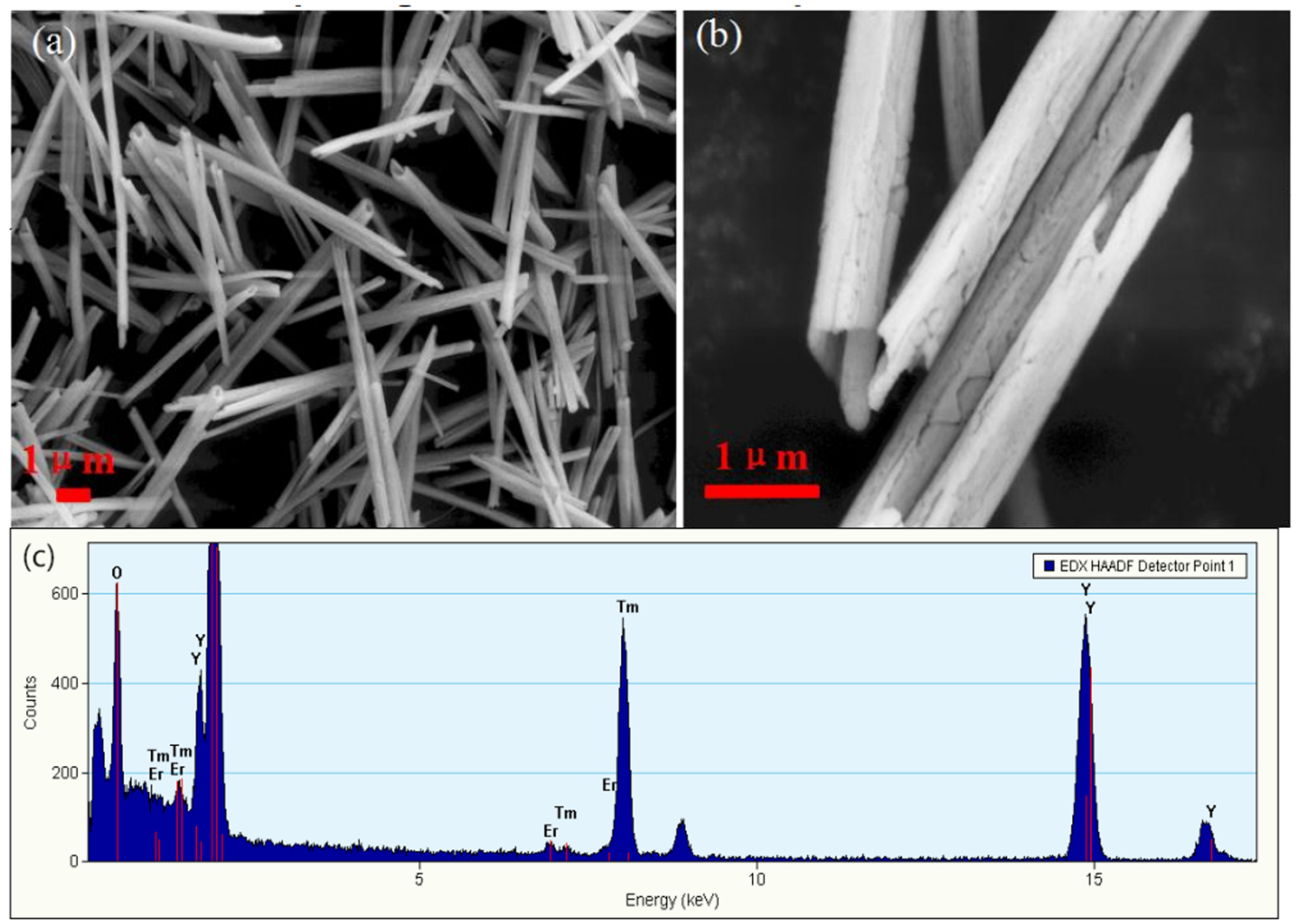
(**a**) SEM image, (**b**) enlarged SEM image, and (**c**) EDS spectrum of Y_2_O_3_:1%Er^3+^, 1.5%Tm^3+^.

**Figure 2:**
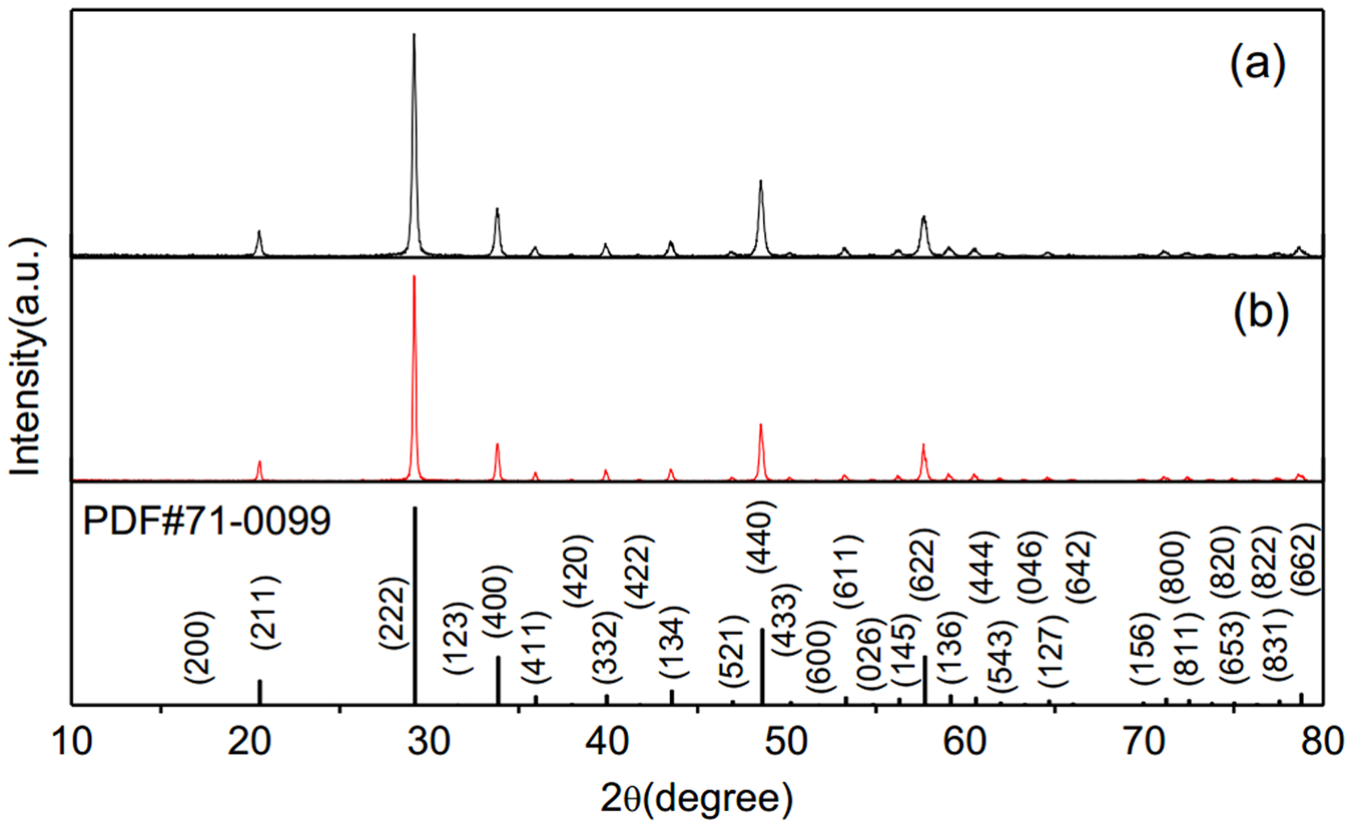
Powder XRD patterns of (**a**) Y_2_O_3_:1%Er^3+^, 1.5%Tm^3+^ and (**b**) Y_2_O_3_:1%Er^3+^, 0.5%Ho^3+^. Below is the standard card.

The low and high-magnification SEM images of Y_2_O_3_:1%Er^3+^, 1.5%Tm^3+^ in Fig. 1(a) and (b) show that the feature of the sample is a hollow and tubular column, and the average diameter is 0.7μm. The EDS spectrum in Fig. 1(c) shows that the samples consist of O, Er, Tm, and Y, which is in good accordance with the initial elements in precursor solution. All the synthesized Y_2_O_3_:1%Er^3+^ and Y_2_O_3_:1%Er^3+^, 0.5%Ho^3+^ microtubes were analyzed ﻿also by the SEM and EDS. It is found that the shapes of all the samples are similar, and their sizes have no obvious change. Figure 2 displays the powders XRD patterns of Y_2_O_3_:1%Er^3+^, 1.5%Tm^3+^ and Y_2_O_3_:1%Er^3+^, 0.5%Ho^3+^ microtubes. The position and relative intensity of all the diffraction peaks can be readily indexed to the pure cubic Y_2_O_3_ according to the JCPDS file no. 71-0099. No peak is recorded from other phases or impurities, which indicates the phase of the sample was pure Y_2_O_3_ tubes. From XRD patterns, one can find that the Y_2_O_3_ microtubes grow along the single direction (222) plane.

**Figure 3:**
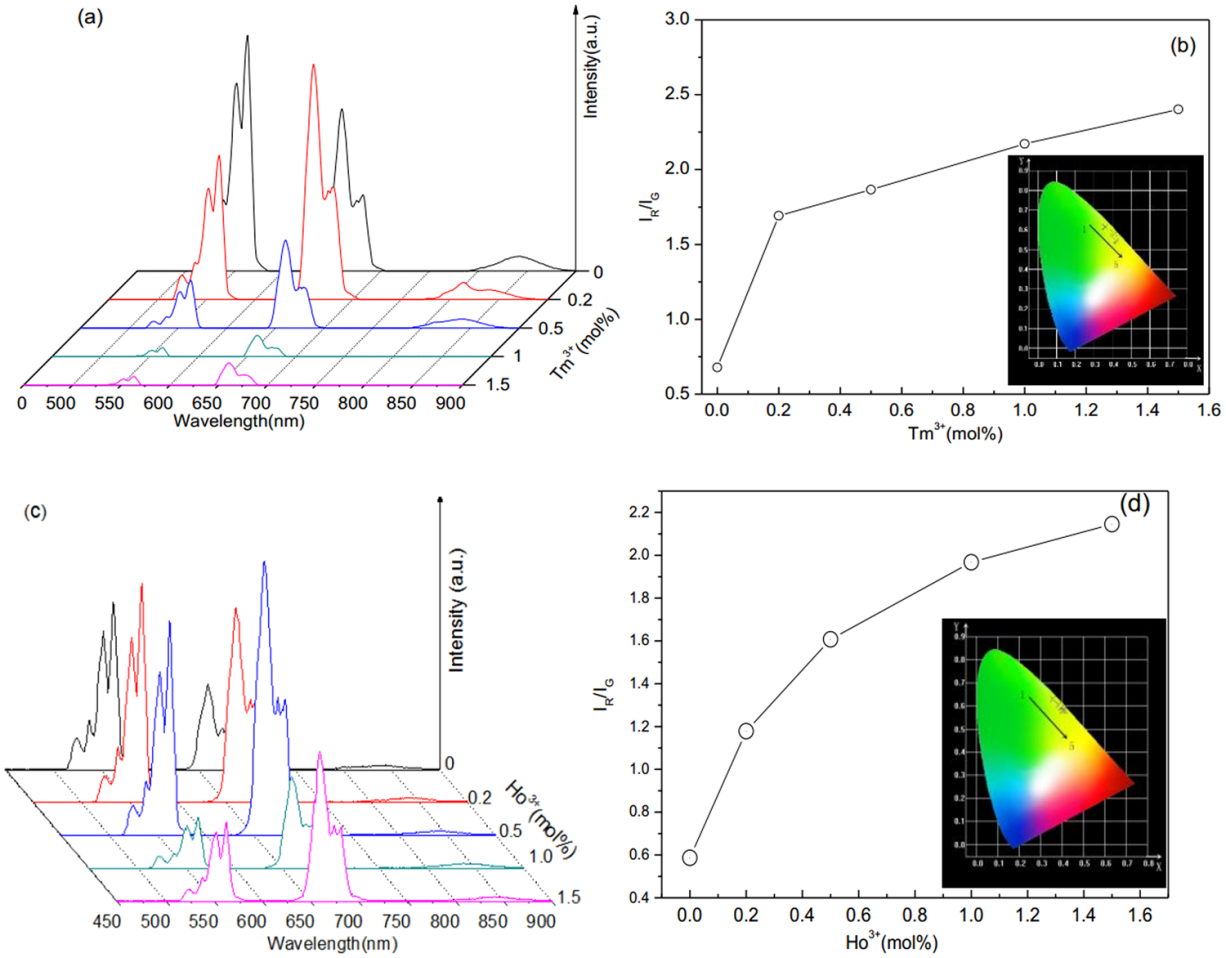
(**a**) Emission spectra, (**b**) red to green intensity ratio and CIE (X, Y) chromaticity coordinates diagram of 1%Er^3+^, x%Tm^3+^ co-doped Y_2_O_3_ (x = 0, 0.2, 0.5, 1, 1.5). (**c**) Emission spectra and (**d**) Red to green intensity ratio and CIE (X, Y) chromaticity coordinates diagram of 1%Er^3+^, x%Ho^3+^ co-doped Y_2_O_3_ (x = 0, 0.2, 0.5, 1, 1.5).

In order to study the role of Tm^3+^ and Ho^3+^ ions, the up-conversion spectra, red to green intensity ratios and CIE (X, Y) chromaticity coordinates of Y_2_O_3_:1%Er^3+^ co-doped Tm^3+^ and Ho^3+^ are given in Fig. 3. Figure 3(a) shows the seven emission bands centered at 524 nm, 537 nm, 552 nm and 660 nm, 680 nm, 812 nm, 843 nm, which corresponds to the ^2^H_11/2_ → ^4^I_15/2_ (524–537 nm), ^4^S_3/2_ → ^4^I_15/2_ (552 nm), ^4^F_9/2_ → ^4^I_15/2_ (660–680 nm), and ^4^I_9/2_ → ^4^I_15/2_ (843 nm) transitions of Er^3+^ ions and ^3^H_4_ → ^3^H_6_ (812 nm) transition of Tm^3+^ ions. It is observed that the luminescence intensity of the green and 840 nm infrared emission decrease with increasing Tm^3+^ concentration, and the intensity of red emission first increases and then decreases. The intensity decrease of the green and red emissions is attributed to the quenching induced by the cross relaxation energy transfer between Tm^3+^ and Er^3+^: ^4^F_7/2_ + ^3^H_6_ → ^4^I_9/2_ + ^3^H_5_^31^. Figure 3(b) shows the red-green intensity ratio increases with the increase of Tm^3+^ concentration, and luminescent color is tunable from green to red with the increase of Tm^3+^ concentration. Similar spectrum modulation and color adjustment can be achieved through co-doping Ho^3+^ into Y_2_O_3_:1%Er^3+^ microtubes, as shown in Fig. 3(c) and (d). The intensities of the green and red emissions show the irregularly change with increasing Ho^3+^ concentration, while the intensity of infrared emission has no obvious change. The intensity increase of the green and red emissions of Y_2_O_3_:1%Er^3+^, when the Ho^3+^ concentration is more than 1%, is attributed to the contribution of Ho^3+^ on green and red emissions through the ^5^S_2_ → ^5^I_8_ (green) and ^5^F_5_ → ^5^I_8_ (red) transitions^33^. The Ho^3+^ doping induces the increase of the red-green intensity ratio, and the enhancement of the yellow color, as shown in Fig. 3(d). After doping with Tm^3+^ and Ho^3+^, the emission intensity, the red-green intensity ratio, and the color of Y_2_O_3_:Er^3+^ are adjusted efficiently. This means that it is possible to adjust the optical temperature behavior of Y_2_O_3_:Er^3+^ at a high temperature through doping Tm^3+^ and Ho^3+^ ions.

**Figure 4:**
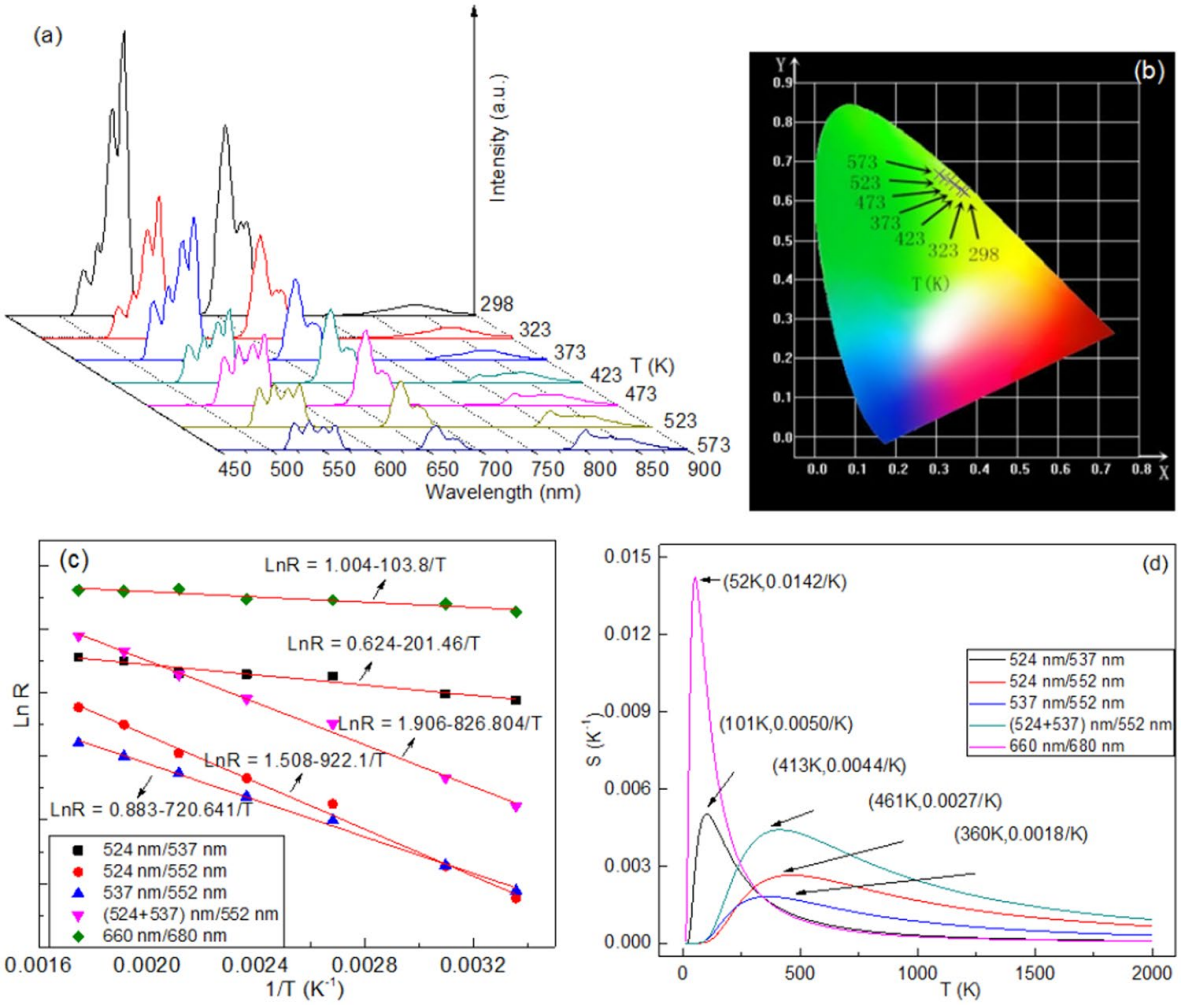
Temperature dependent (**a**) emission spectra, (**b**) CIE (X, Y) chromaticity coordinates diagram, (**c**) Arrhenius plots of emission intensity ratios, and (**d**) sensitivity of Y_2_O_3_:1%Er^3+^at 184 mW/mm^2^ excitation.

Temperature-dependent emission spectra of Y_2_O_3_:1%Er^3+^ is shown in Fig. 4(a). It can be seen that the red and green emissions continuously decrease by increasing the temperature from 298 K to 573 K, without changing the peak positions of the emissions. The color shifting from yellow to green in Fig. 4(b) indicates the inhomogeneous decrease of green and red emissions induced by the high temperature. It is necessary to study the dependence of the fluorescence intensity ratio (*R*) on the temperature for the adjacent emission bands. The relation between *R* and *T* is expressed as:

1 $$LnR=-a/T+b$$

where *a* is constant, *b* is a correction term for the comprehensive population of thermally coupled energy levels induced by the thermal population, nonradiative relaxation and so on^2, 34^. Relative sensitivity is one of the key parameters to determine the suitability for optical thermometry, and is defined as

2 $$S=\frac{dR}{dT}=\frac{a}{{T}^{2}}{e}^{\frac{bT-a}{T}}$$

where *a* and *b* are the constants from Eq. (1). Figure 4(c) shows temperature-dependent emission intensity ratios of several adjacent emission bands. The experimental data points can be fitted well with a line model. The slope values of fitted lines are dependent on the combination types of adjacent emission bands. It means that the sensitivity values are different when we use the different adjacent emission bands as the thermal coupled levels. Figure 4(d) shows the temperature dependent sensitivity values of five thermal coupled levels. One can find that all the sensitivity values increase and then decrease with the temperature increase, exhibiting the maxima values at different temperature points. The maximum values at (101 K, 0.0050/K), (461 K, 0.0027/K), (360 K, 0.0018/K), (413 K, 0.0044/K), (52 K, 0.0142/K) are observed for the adjacent thermal coupled levels of 524 nm/537 nm, 524 nm/552 nm, 537 nm/552 nm, (524 + 537) nm/552 nm, 660 nm/680 nm. Notably, the adjacent thermal coupled levels of (524 + 537) nm/552 nm shows the large intensity in the high temperature range. The adjacent thermal coupled levels of 660 nm/680 nm shows the very large intensity in the low temperature range. As reported, the Er^3+^-Yb^3+^ co-doped Y_2_O_3_ sphere nano-particles showed the maximum sensitivity value of 0.0044/K at 427 K^28^, and the Er^3+^-Yb^3+^-Eu^3+^ tri-doped Y_2_O_3_ sphere nanoparticles showed the maximum sensitivity value of 0.0103/K at 593 K^27^. In contrast, our Er^3+^- doped Y_2_O_3_ microtubes show a large sensitivity value of 0.0142/K in the low temperature range, and an excellent sensitivity value of 0.0044/K in the high temperature range. Most of the sensors based on up-conversion luminescence of Er^3+^ ion showed excellent sensitivity properties at the high temperature of more than 300 K^12, 14^, while it was reported rarely on optical thermometry below room temperature.

**Figure 5:**
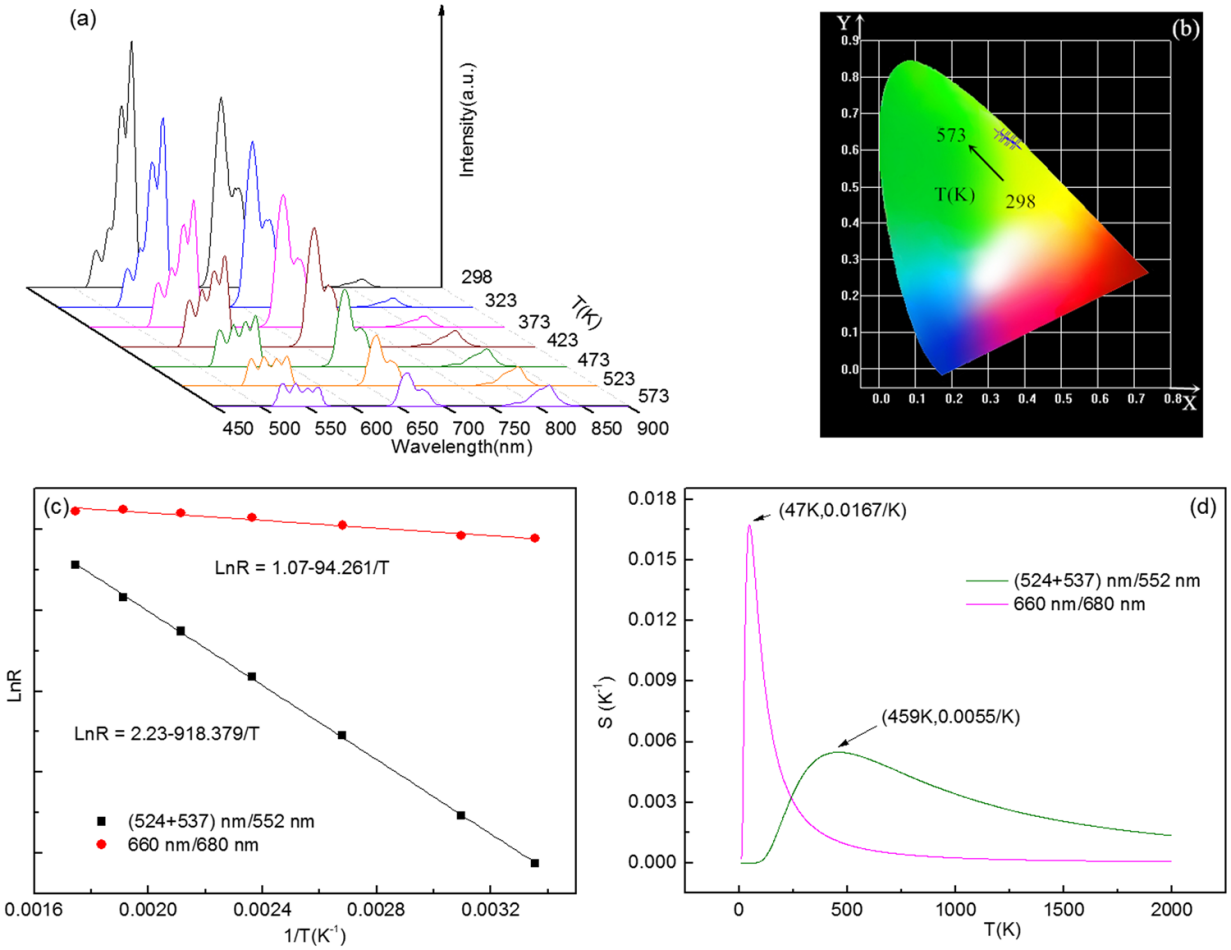
Temperature dependent (**a**) emission spectra, (**b**) CIE (X, Y) chromaticity coordinates diagram, (**c**) Arrhenius plots of emission intensity ratios, and (**d**) sensitivity of Y_2_O_3_:1%Er^3+^, 0.2%Tm^3+^ at 184 mW/mm^2^ excitation.

**Figure 6:**
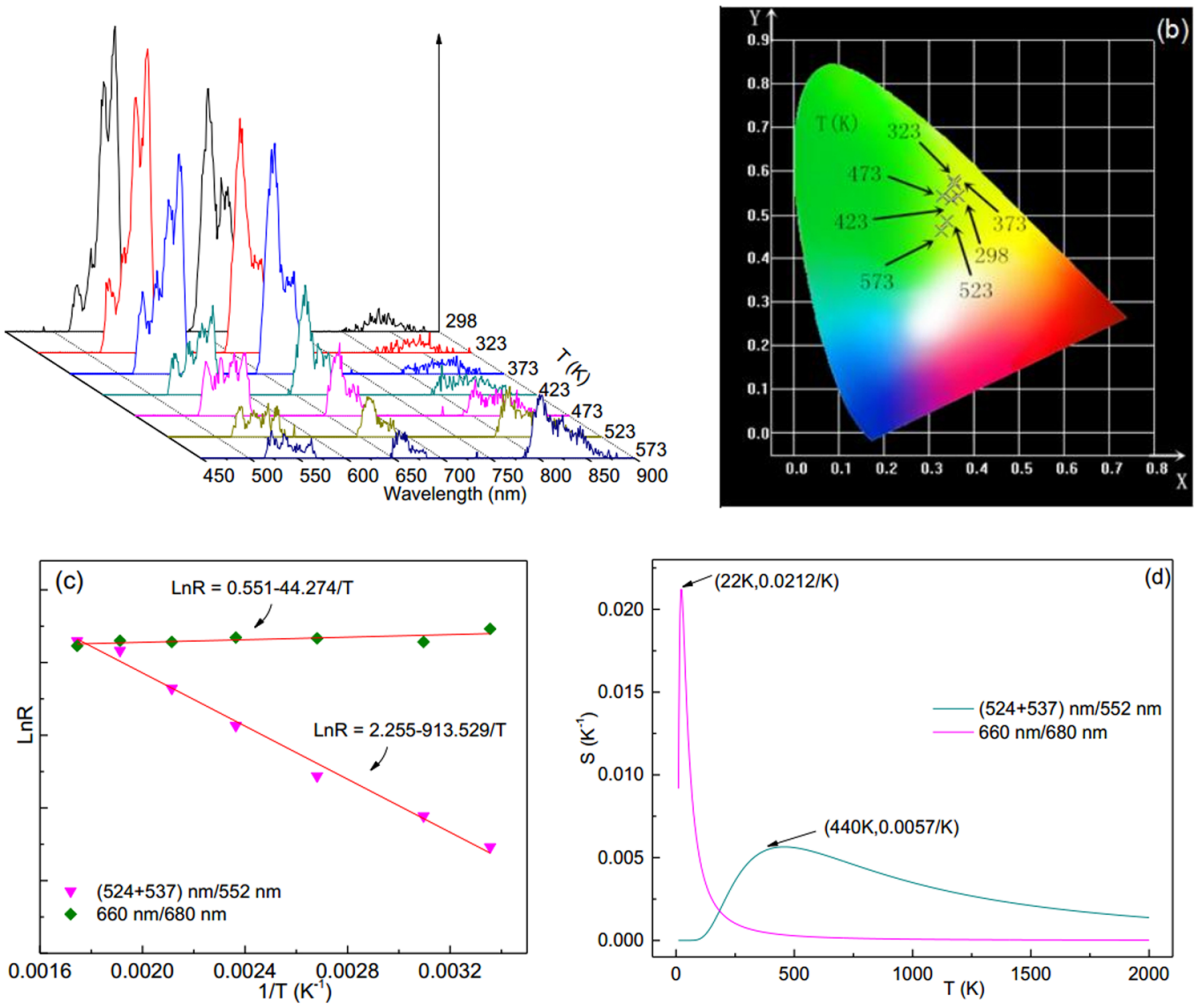
Temperature dependent (**a**) emission spectra, (**b**) CIE (X, Y) chromaticity coordinates diagram, and (**c**) Arrhenius plots of emission intensity ratios, and (**d**) sensitivity of Y_2_O_3_:1%Er^3+^, 0.5%Ho^3+^ at 184 mW/mm^2^ excitation.

The influence of Tm^3+^ and Ho^3+^ on the optical temperature behaviors of Y_2_O_3_:1%Er^3+^ is studied through co-doping 0.2 mol% Tm^3+^ and 0.5 mol% Ho^3+^ into Y_2_O_3_:1%Er^3+^, as shown in Figs 5 and 6. Compared with the Fig. 4, it is evident that the emission spectra, CIE chromaticity coordinates, and *R* values of Y_2_O_3_:1%Er^3+^ are adjusted after co-doping with Tm^3+^ or Ho^3+^. Importantly, after co-doping with Tm^3+^ and Ho^3+^ ions, the maximum sensitivity value based on the thermal coupled levels of (524 + 537) nm/552 nm, is greatly enhanced and shifts to the higher temperature range, while the maximum sensitivity value based on the thermal coupled levels of 660 nm/680 nm, shifts to lower temperature range and increases a lot. This makes it possible to achieve the optical temperature measurement in the low temperature range.

**Figure 7:**
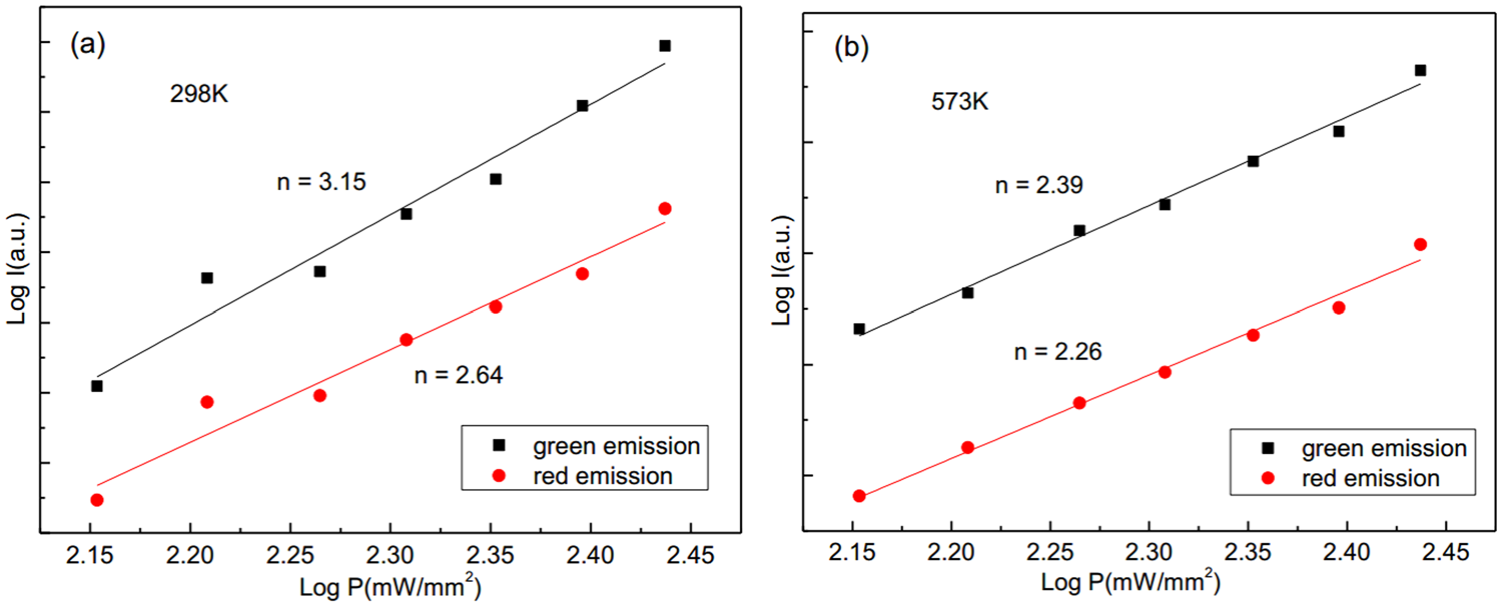
Log-log plots of emission intensity and pumping power for Y_2_O_3_:1%Er^3+^ at (**a**) 298 K and (**b**) 573 K.

**Figure 8:**
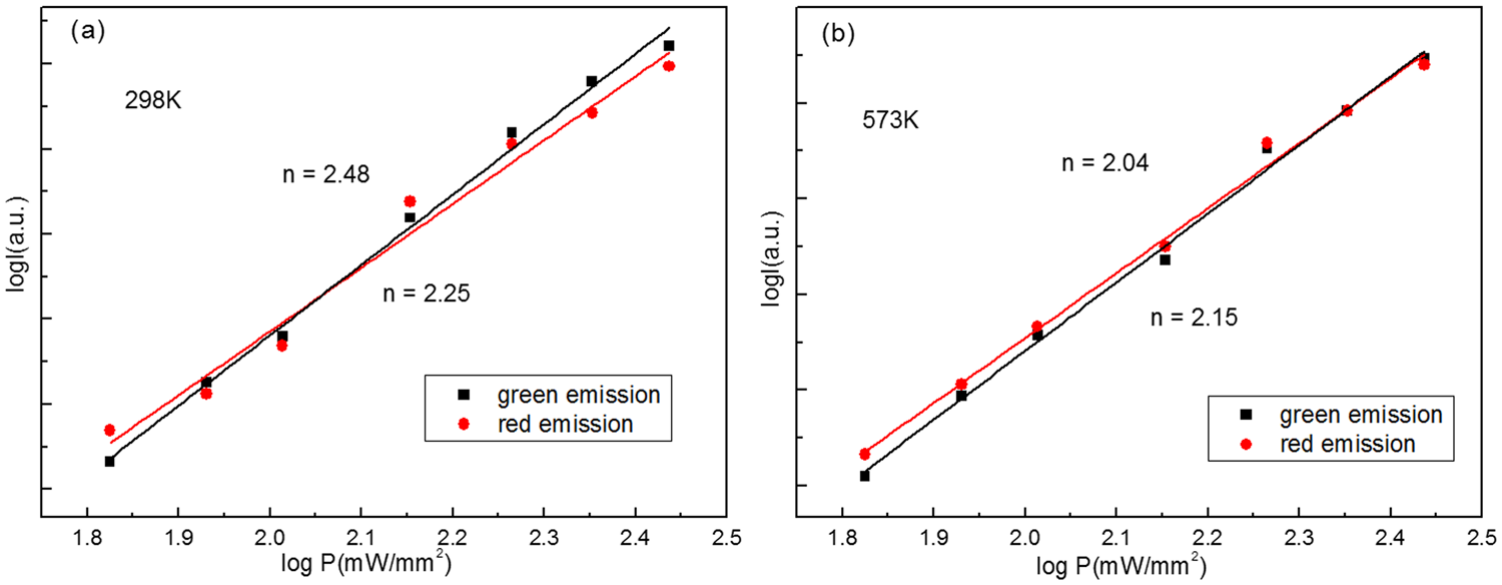
Log-log plots of emission intensity and pumping power for Y_2_O_3_:1%Er^3+^, 0.2%Tm^3+^ at (**a**) 298 K and (**b**) 573 K.

**Figure 9:**
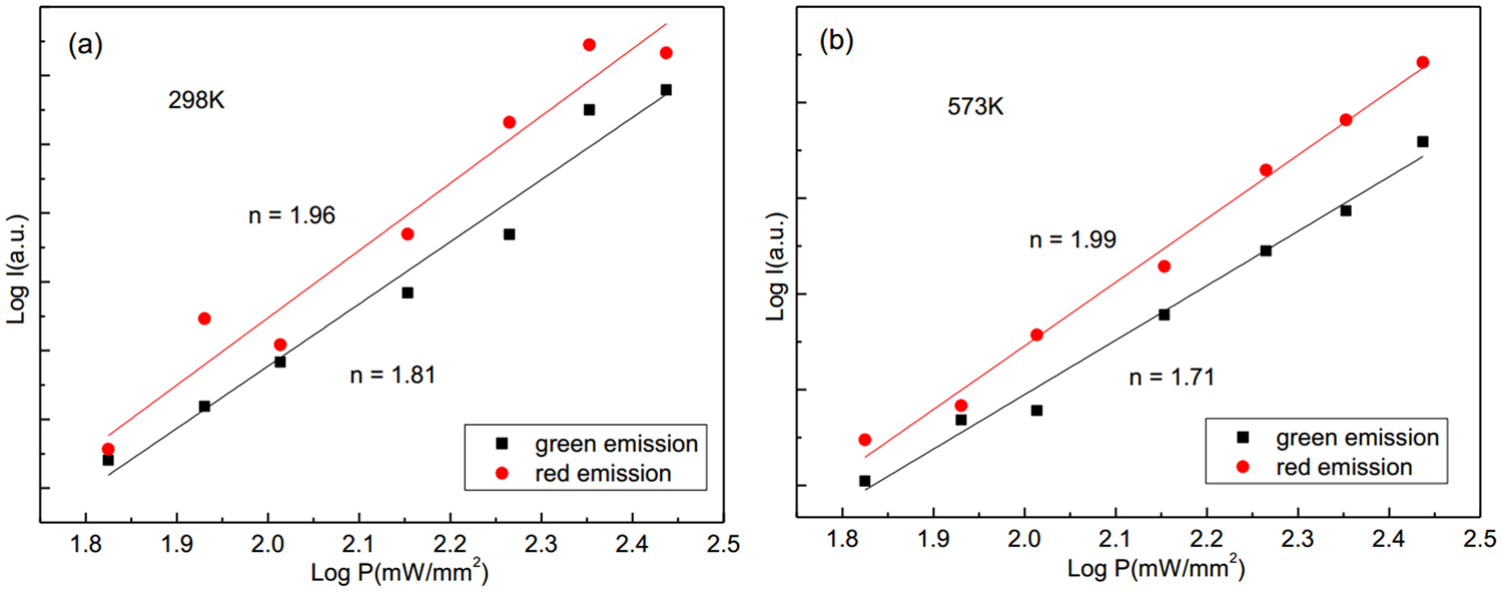
Log-log plots of emission intensity and pumping power for Y_2_O_3_:1%Er^3+^, 0.5%Ho^3+^ at (**a**) 298 K and (**b**) 573 K.

It is necessary to study the thermal stability of thermal coupled levels in the process of optical temperature sensing. The thermal stability of emission bands can be determined by the number change of photons involved in the up-conversion processes at a different temperature. The up-conversion emission intensity *I* and excitation power *P* is expressed as follows:

3 $$I\propto {P}^{n}$$

where *I* is the emission intensity, *P* is incident pump power, and *n* is the number of pump photons absorbed in the up-conversion process^35^. Figure 7 shows the double logarithmic plots of the emission intensity *I* as a function of pump power *P* of the Y_2_O_3_:Er^3+^. The fit results indicate that three infrared photons are needed to emit green and red luminescence at 298 K and 573 K. After doping Tm^3+^ and Ho^3+^ into Y_2_O_3_:Er^3+^, the fit results indicate that two infrared photons are needed to emit green and red luminescence at 298 K and 573 K, as shown in Figs 8 and 9. The decrease of slope values for green and red emissions means that the up-conversion process becomes easy to occur at the same excitation power. Thus, the thermal stability of Y_2_O_3_:Er^3+^ microtubes is improved through co-doping Tm^3+^ and Ho^3+^ ions.

**Figure 10:**
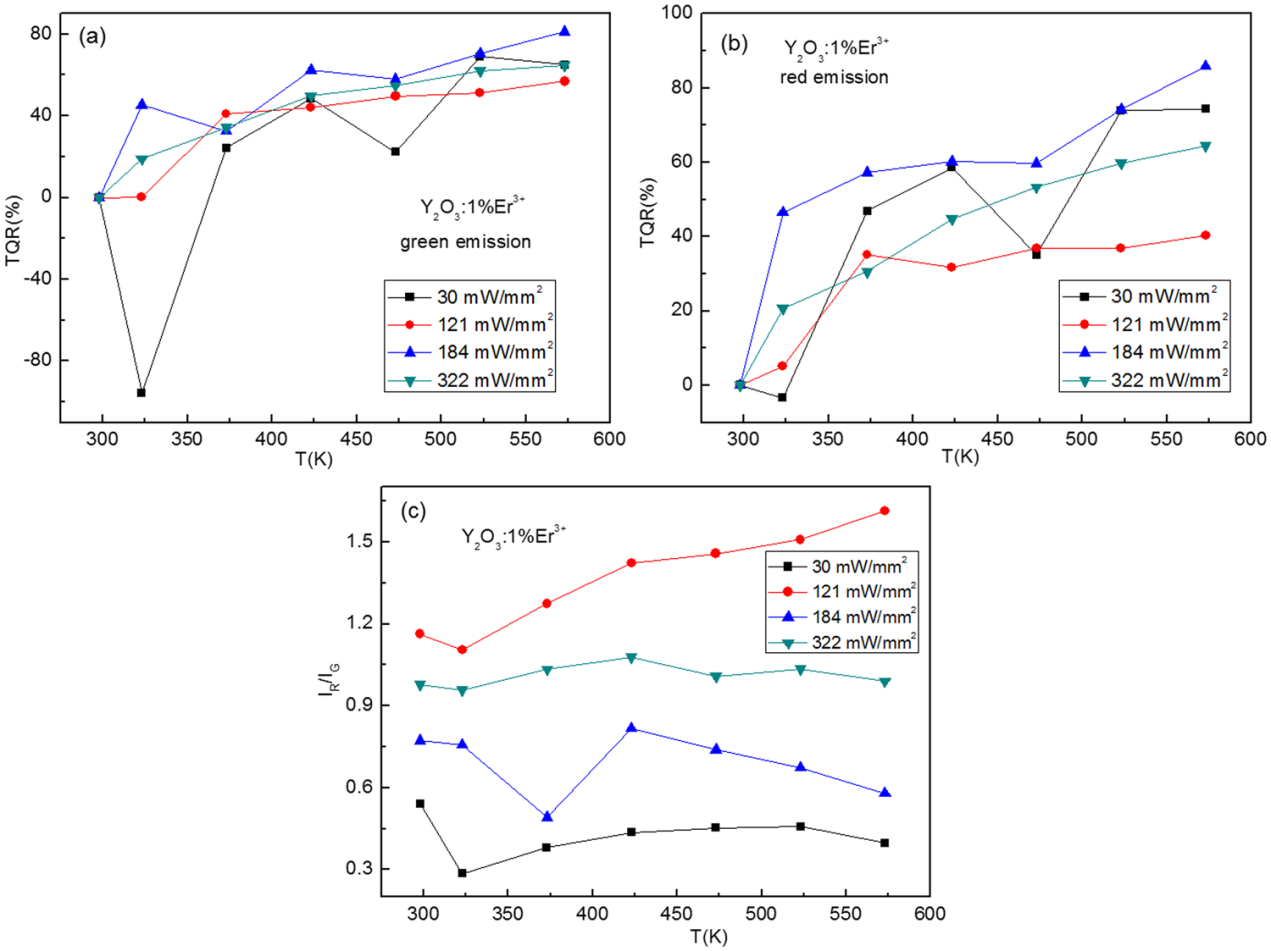
Excitation powers dependent *TRQ* of (**a**) green and (**b**) red emissions of Y_2_O_3_:1%Er^3+^, and (**c**) red to green intensity ratio of Y_2_O_3_:1%Er^3+^.

**Figure 11:**
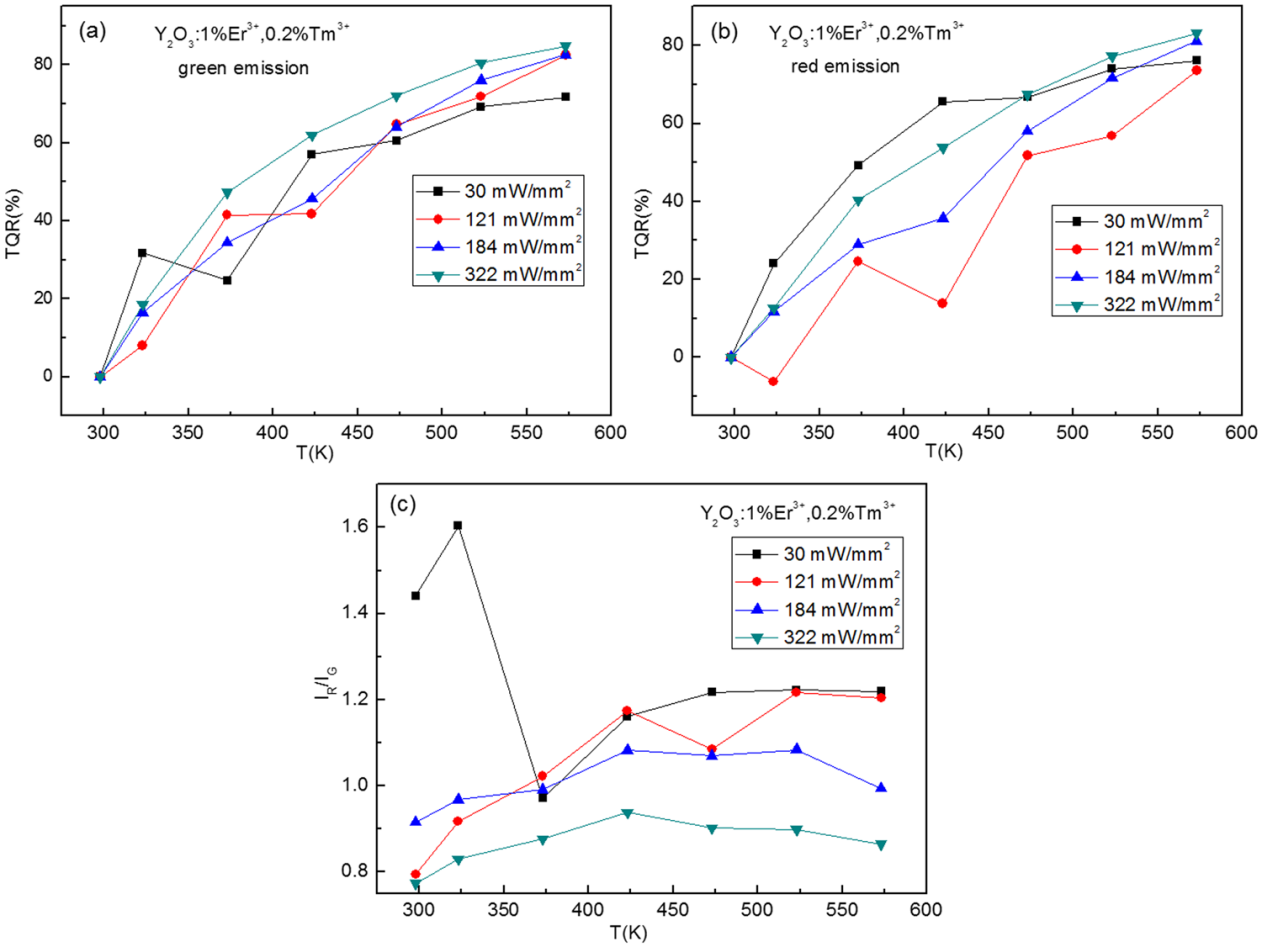
Excitation powers dependent *TRQ* of (**a**) green and (**b**) red emissions of Y_2_O_3_:1%Er^3+^, 0.2%Tm^3+^, and (**c**) red to green intensity ratio of Y_2_O_3_:1%Er^3+^, 0.2%Tm^3+^.

**Figure 12:**
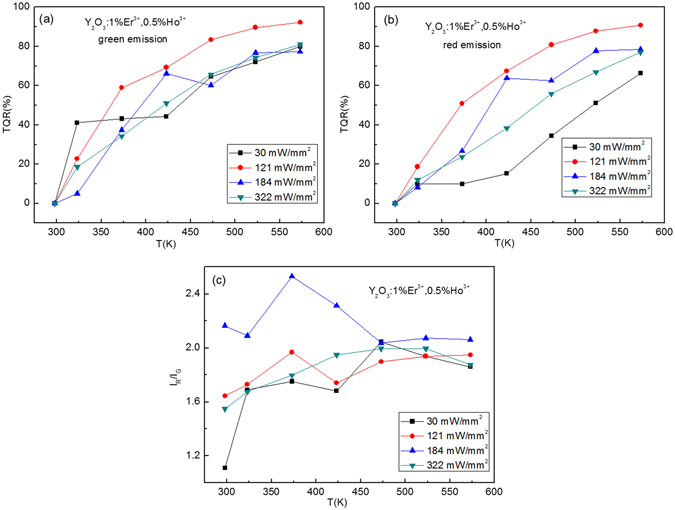
Excitation powers dependent *TRQ* of (**a**) green and (**b**) red emissions of Y_2_O_3_:1%Er^3+^, 0.5%Ho^3+^, and (**c**) red to green intensity ratio of Y_2_O_3_:1%Er^3+^, 0.5%Ho^3+^.

M. Pollnau observed that the population processes of green and red emissions were adjusted by the excitation power density^35^. It is necessary to study the excitation power-dependent optical temperature behaviors of Y_2_O_3_:1%Er^3+^, Y_2_O_3_:1%Er^3+^, 0.2%Tm^3+^, and Y_2_O_3_:1%Er^3+^, 0.5%Ho^3+^. To evaluate the influence of excitation powers on luminescence quenching, the thermal quenching ratio (*TRQ*) of Er^3+^ emission bands at different excitation powers are studied in Figs 10, 11 and 12. The thermal quenching ratio (*TRQ*) of emission bands induced by the temperature change is defined as follows

4 $$TRQ=1-\frac{{I}_{T}}{{I}_{R}}$$

where *I*_*T*_ is luminescence intensity at a different temperature *T*, and *I*_*R*_ is luminescence intensity at room temperature^36^. From Fig. 10(a) and (b), it is obvious that the *TRQ* of green and red emissions of Y_2_O_3_:1%Er^3+^ is strongly dependent on temperature and excitation powers. The values of *TRQ* of green and red emissions increase with the increase of temperature, change irregularly with the increase of excitation powers. The red-to-green intensity ratio is dependent on both temperature and excitation powers, and shows the large values at the excitation power of 121 mW/mm^2^, as shown in Fig. 10(c). After co-doping with the Tm^3+^ and Ho^3+^, the *TRQ* values of green and red emissions and red-to-green emission intensity ratios of Y_2_O_3_: 1%Er^3+^ are adjusted, as shown in Figs 11 and 12.

**Figure 13:**
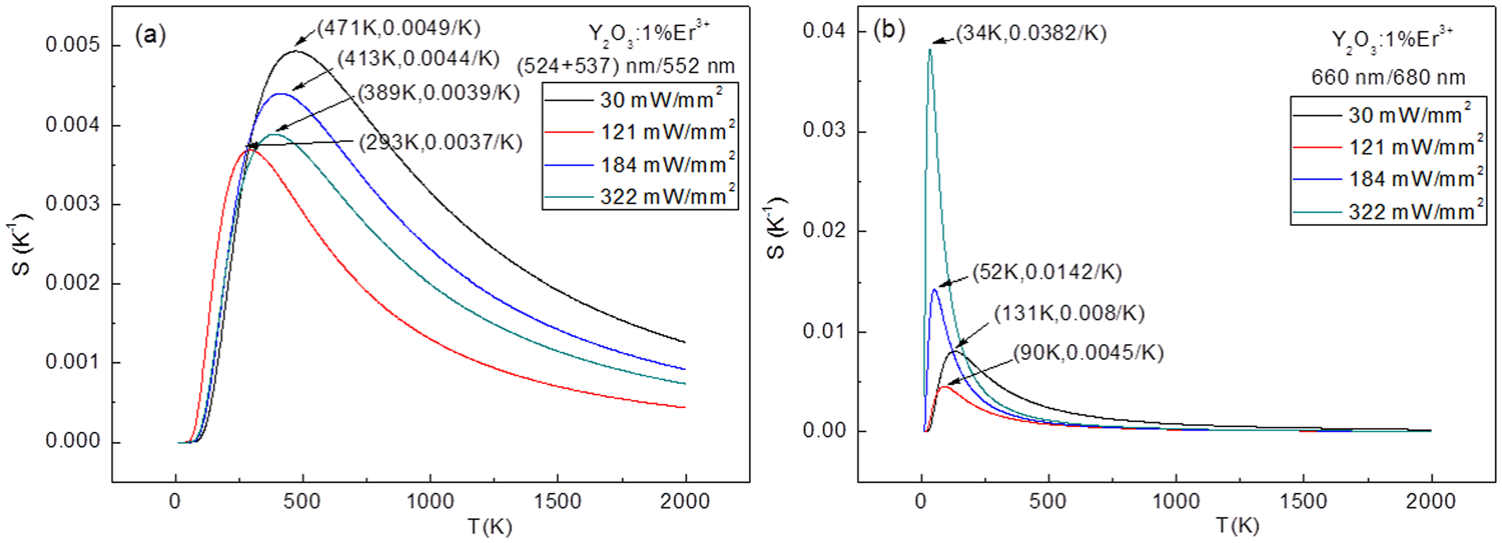
Excitation powers dependent sensitivity of Y_2_O_3_:1%Er^3+^.

**Figure 14:**
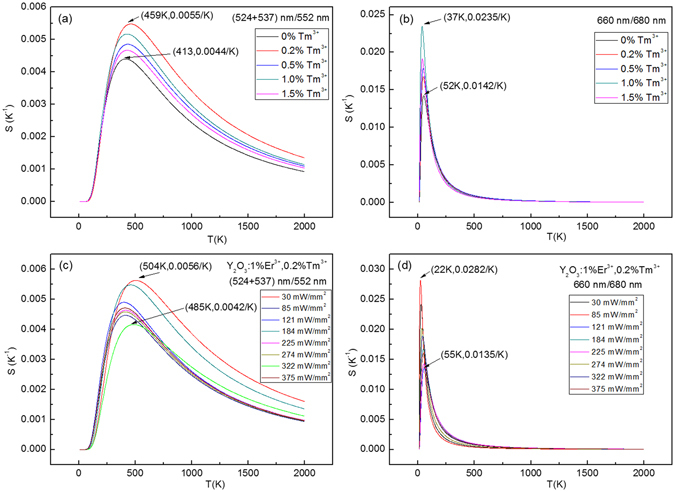
Doped concentration dependent sensitivity of Y_2_O_3_:1%Er^3+^, x%Tm^3+^ (x = 0, 0.2, 0.5, 1.0, 1.5), and excitation powers dependent sensitivity of Y_2_O_3_:1%Er^3+^ 0.2%Tm^3+^.

**Figure 15:**
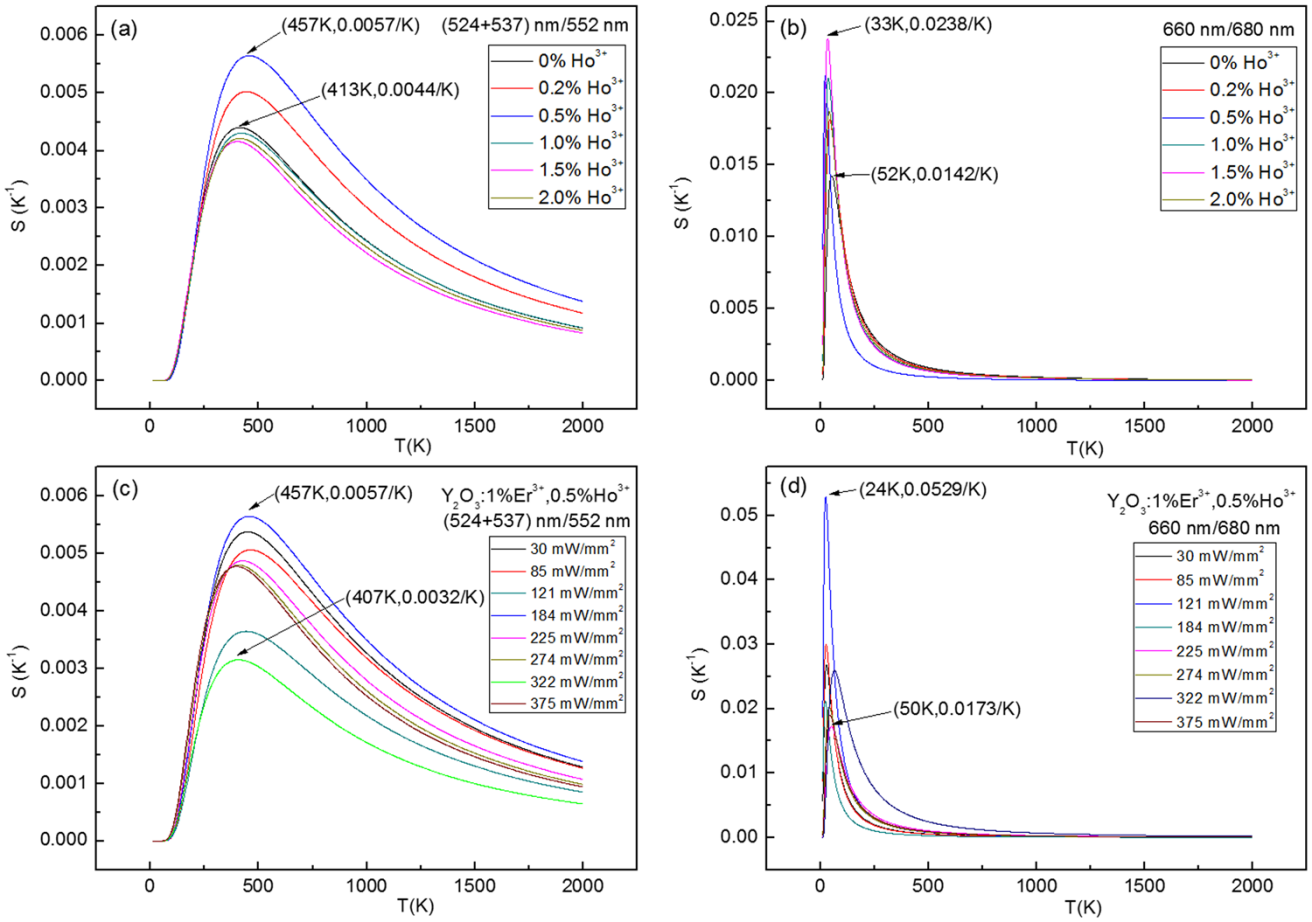
Doped concentration dependent sensitivity of Y_2_O_3_:1%Er^3+^, y%Ho^3+^ (x = 0, 0.2, 0.5, 1.0, 1.5, 2.0), and excitation powers dependent sensitivity of Y_2_O_3_:1%Er^3+^, 0.5%Ho^3+^.

The influence of excitation powers on the sensitivity values for the Y_2_O_3_:1%Er^3+^ are studied in Fig. 13. At different excitation powers, based on the ratio of (524 + 537) nm/552 nm, the maximum sensitivity value of 0.0049/K at 471 K is achieved at 30 mW/mm^2^, as shown in Fig. 13(a). Based on the ratio of 660 nm/680 nm, the maximum sensitivity value of 0.0382/K at 34 K is achieved at 322 mW/mm^2^, as shown in Fig. 13(b). After co-doping with Tm^3+^, the maximum sensitivity values are significantly enhanced and shift to the high temperature range with various Tm^3+^ concentrations, as shown in Fig. 14(a) and (b). The sensitivity value based on the ratio of (524 + 537) nm/552 nm achieves the maximum value at (459 K, 0.0055/K) when the Tm^3+^ concentration is 0.2 mol%. The sensitivity value based on the ratio of 660 nm/680 nm achieve the maximum value at (37 K, 0.0235/K) when the Tm^3+^ concentration is 1.0 mol%. Furthermore, the excitation power dependent sensitivity is studied in Fig. 14(c) and (d). The maximum sensitivity values change irregularly with the increase of the excitation powers, and reach the maximum value of (504 K, 0.0056/K) at 30 mW/mm^2^ for the ratio of (524 + 537) nm/552 nm, and the maximum value of (22 K, 0.0282/K) at 85 mW/mm^2^ for the ratio of 660 nm/680 nm. Similarly, the influence of Ho^3+^ concentration and excitation powers on the sensitivity values are also studied in Fig. 15. The optimized Ho^3+^ concentration is obtained is 0.5 mol%. For the Y_2_O_3_:1%Er^3+^, 0.5%Ho^3+^, the maximum sensitivity values change irregularly with the increase of the excitation powers, and reach the maximum value of (457 K, 0.0057/K) at 184 mW/mm^2^ for the ratio of (524 + 537) nm/552 nm, and the maximum value of (24 K, 0.0529/K) at 121 mW/mm^2^ for the ratio of 660 nm/680 nm. Compared with the Y_2_O_3_:1%Er^3+^, the maximum sensitivity value increases from 0.0049/K (0.0382/K) to 0.0057/K (0.0529/K) through co-doping with the 0.5 mol% Ho^3+^ ions. In order to compare the sensitivity values of Y_2_O_3_ doped with other ions for the fluorescence thermometric study, the reported sensitivity values based on the up-conversion fluorescence of Er^3+^ are listed in Table S1 in the supplementary information. It can be seen that the sensitivity values in this paper are higher than that of other Y_2_O_3_ materials not only in the high temperature range but also in the low temperature range. It means that it is a good method to improve the sensitivity of Y_2_O_3_:Er^3+^ through co-doping Ho^3+^ ions.

**Figure 16:**
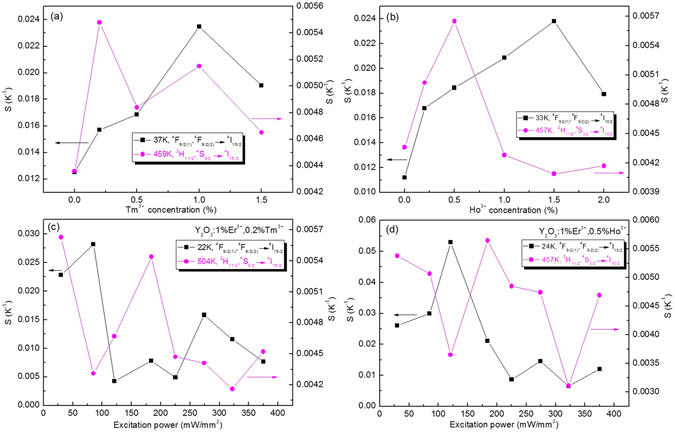
Doping concentration and excitation powers dependent sensitivity of Y_2_O_3_:1%Er^3+^ 0.2%Tm^3+^ and Y_2_O_3_:1%Er^3+^, 0.5%Ho^3+^ samples.

The influence of doping concentration and excitation powers on the sensitivity values for the Y_2_O_3_:1%Er^3+^ are also studied at the fixed temperatures, as shown in Fig. 16. One can find that the sensitivity values from the ^4^F_9/2(1)_/^4^F_9/2(2)_ thermal coupled levels increase and then decrease with the increase of doping concentrations of Tm^3+^ and Ho^3+^ ions, while the sensitivity values from the ^2^H_11/2_/^4^S_3/2_ thermal coupled levels change irregularly with the increase of doping concentrations, as shown in Fig. 16(a) and (b). The optimized Tm^3+^ and Ho^3+^ concentrations are 0.2 mol% and 0.5 mol%, respectively. The sensitivity values from the ^2^H_11/2_/^4^S_3/2_ and ^4^F_9/2(1)_/^4^F_9/2(2)_ thermal coupled levels show several oscillating curves with the increase of excitation powers, as shown in Fig. 16(c) and (d). For the Y_2_O_3_:1%Er^3+^, 0.2%Tm^3+^, the sensitivity value based on ^2^H_11/2_/^4^S_3/2_ thermal coupled levels reaches the maximum value at 30 mW/mm^2^, and the sensitivity value based on ^4^F_9/2(1)_/^4^F_9/2(2)_ thermal coupled levels reaches the maximum value at 85 mW/mm^2^, as shown in Fig. 16(c). For the Y_2_O_3_:1%Er^3+^, 0.5%Ho^3+^, the sensitivity value based on ^2^H_11/2_/^4^S_3/2_ thermal coupled levels reaches the maximum value at 184 mW/mm^2^, and the sensitivity value based on ^4^F_9/2(1)_/^4^F_9/2(2)_ thermal coupled levels reaches the maximum value at 121 mW/mm^2^, as shown in Fig. 16(d).

To study the influence of doping concentration and excitation powers on the optical temperature behaviors, the dynamic balance rate-equation models for the energy transfer between Er^3+^ and Tm^3+^(or Ho^3+^) are established in Figs S1 and S2 in the supplementary information. The population dynamic process of excited states is simulated by using the eight-level model. The population density of the ^2^H_11/2_/^4^S_3/2_ and ^4^F_9/2(1)_/^4^F_9/2(2)_ thermal coupled levels can be obtained in the Equations S(8), S(9), S(17), and S(18). It is obvious that the values of *N*_*3*_ and *N*_*4*_ are dependent on not only the excitation power (ρ) and doping concentration (N_0_, N_6_), but also the cross relaxation rates (*W*_c1_, *W*_c2_, *W*_c3_, and *W*_c4_), nonradiative decay rates (*W*_*ij*_), and radiative transition rates (*A*_*ij*_). Notably, the cross relaxation rates, nonradiative decay rates, and radiative transition rates are strongly dependent on the temperature^37^. It means that the population processes of the ^2^H_11/2_/^4^S_3/2_ and ^4^F_9/2(1)_/^4^F_9/2(2)_ thermal coupled levels are determined by the excitation power, doping concentration and temperature. It is a complex multi-field coupling effect. Thus, the sensitivity shows irregularly change with the increase of excitation powers and doping concentrations.

Briefly speaking, at low excitation power, the thermal coupled levels of ^4^S_3/2_ and ^2^H_11/2_ are populated by the ground state absorption (GSA) and excited state absorption (ESA), and then the multiphonon nonradiative relaxation (NR) from ^4^F_7/2_ level, shown in Fig. S1. The thermal coupled levels of ^4^F_9/2(1)_ and ^4^F_9/2(2)_ are populated by the NR process from the ^4^S_3/2_ level. According to the Boltzmann distribution^14, 15^, the population of the ^2^H_11/2_ level increases with respect to the ^4^S_3/2_ with the increase of the temperature, owe to the low energy difference between ^4^S_3/2_ and ^2^H_11/2_ levels. The thermalization of the ^2^H_11/2_ level is dominant and the depopulation induced by the NR process can be neglected. At high excitation power, the population saturation effect of the ^4^S_3/2_ and ^2^H_11/2_ levels can be observed^35, 38^. Therefore, the population of ^2^H_11/2_ level changes a little with the temperature. The NR is easy to occur in the case of small energy difference between adjacent energy levels at the high temperature^19, 20^. The NR process is dominant to depopulate the ^2^H_11/2_ level, due to the small energy difference between ^2^H_11/2_ and ^4^S_3/2_ (*ΔE* = 968 cm^−1^). Thus, the R in equation 1 decreases with the increase of excitation power density, due to the fact that the NR possibility increases with the temperature increase. As a result, the sensitivity decreases at high excitation power density. Importantly, after co-doping Tm^3+^ and Ho^3+^ ion, the sensitivity values shift to higher and lower temperature ranges, and are greatly increased. It is attributed to the fact that the populations of ^4^S_3/2_/^2^H_11/2_ and ^4^F_9/2(1)_/^4^F_9/2(2)_ levels are adjusted by the cross relaxation process, such as CR1 and CR3. With the concentration further increase of the Tm^3+^ and Ho^3+^ ions, the green and red emissions are quenched, due to the cross relaxation process, such as CR2 and CR4.

## Conclusions

In summary, we explore a method to improve the photoluminescence and optical temperature sensing of Er^3+^ doped Y_2_O_3_ microtubes through combining the ion doping with the control of excitation powers. It is found that the optical temperature behaviors of Er^3+^ doped Y_2_O_3_ microtubes are strongly dependent on the ion doping and excitation powers. After doping Tm^3+^ or Ho^3+^ ions, the spectrum of Er^3+^ doped Y_2_O_3_ microtubes is modified, the thermal quenching behavior of Y_2_O_3_:Er^3+^ microtubes is inhibited, and the optical temperature sensitivity is significantly enhanced. It is achieved that the maximum sensitivity value is 0.0529/K at low temperature range of less than 250 K while it is 0.0057/K at more than 250 K by controlling the excitation power to 121 mW/mm^2^. The maximum sensitivity value of 0.0529/K at 24 K is superior to that of our earlier report. It makes up the lack of optical temperature detection in the low temperature range below 250 K.

## Methods

All starting materials are Y_2_O_3_ (99.99%), Er_2_O_3_ (99.99%), Tm_2_O_3_ (99.99%), Ho_2_O_3_ (99.99%), hydrochloric acid (AR), NaOH (AR), ethanol (AR). All chemicals were used without further treatment, and deionized water was used for all experiments.

The Re_2_O_3_ (Re = Er, Y, Tm, and Ho) was dissolved in hydrochloric acid, and then the solution was heated to evaporate the water completely. The obtained rare earth metal trichloride (ReCl_3_) was dissolved in deionized water to prepare the solutions of ReCl_3_ (0.2 mol L^−1^). Er^3+^ doped Y_2_O_3_ microtubes were prepared by a hydrothermal method. In a representative synthesis process, an aqueous solution of 9.90 mL YCl_3_ (0.2 mol L^−1^), 0.10 mL ErCl_3_ (0.2 mol L^−1^) was mixed with 28 mL of distilled water under thorough stirring. It was then vigorously stirred by a magnetic stirrer at room temperature for 30 min, while 3.5 mL of a 5 mol L^−1^ NaOH solution was slowly added in drops. The colloidal solution was then transferred into an Teflon vessel at 473 K for 24 h. The final products were collected, washed several times with ethanol, and purified by centrifugation. Samples were then dried in an oven for 6 h at 373 K. Finally, the dried powders were sintered at 1173 K for 3 h in an electric annealing furnace. After then, the samples were cooled down rapidly to room temperature. The same method was used for Er^3+^/Tm^3+^ and Er^3+^/Ho^3+^ co-doped Y_2_O_3_ microtubes.

The structure of the sample was investigated by X-ray diffraction (XRD) using X’TRA (Switzerland ARL) equipment provided with Cu tube with Kα radiation at 1.54056 Å. The size and shape of the sample were observed by a JSM-IT300 scanning electron microscope (SEM) (JEOL Ltd., Tokyo, Japan) equipped with an energy dispersive X-ray spectrometer (EDS). Luminescence spectra were obtained by the Acton SpectraPro Sp-2300 Spectrophotometer with a photomultiplier tube equipped with a 980 nm laser as ﻿the﻿ excitation source. Different temperature spectra were obtained in the range 298–573 K by using an INTEC HCS302 Hot and Cold System.

## Electronic supplementary material

Supplementary Information
