# Supplementary material for: Improving Optical Temperature Sensing Performance of Er3+ Doped Y2O3 Microtubes via Co-doping and Controlling Excitation Power
Source: Sci Rep. 2017 Apr 7;7:758. doi: 10.1038/s41598-017-00838-w (PMC5429693; doi:10.1038/s41598-017-00838-w)
Supplement: Supplementary file 1 — Supplementary Information [file 41598_2017_838_MOESM1_ESM.pdf]

Supplementary Information

Improving Optical Temperature Sensing Performance of Er^{3+} Doped Y_2O_3 Microtubes via Co-doping and Controlling Excitation Power

Xiangfu Wang,[a*] Ye Wang,[a] Jose Marques-Hueso,[b] and Xiaohong Yan[acd*]

[a]College of Electronic Science and Engineering, Nanjing University of Posts and Telecommunications, Nanjing, 210046, People's Republic of China

[b]Institute of Sensors, Signals and Systems, School of Engineering and Physical Sciences, Heriot-Watt University, Edinburgh, EH14 4AS, United Kingdom

[c]School of Material Science and Engineering, Jiangsu University, Zhenjiang, 212013, People's Republic of China

[d]College of Science, Nanjing University of Aeronautics and Astronautics, Nanjing, 211106, People's Republic of China

***Correspondence to: xfwang@njupt.edu.cn (Wang), yanxh@njupt.edu.cn (Yan)**

Table S1. The maximum sensitivity values of rare earth ions doped Y_2O_3

Rare-earth ions	Host	λ_{ex} (nm)	λ_{em} (nm)	Transitions	S_R(maximum)	Ref
Er^{3+},Yb^{3+}	Y_2O_3 nanodisk	980	528,556	$^2H_{11/2}$,$^4S_{3/2}$→$^4I_{15/2}$	0.0044/K (427 K)	28
Er^{3+},Yb^{3+}	Y_2O_3 nanosphere	978	539,564	$^2H_{11/2}$,$^4S_{3/2}$→$^4I_{15/2}$	0.0528/K (150 K)	29
Er^{3+},Yb^{3+},Eu^{3+}	Y_2O_3 nanodisk	980	523,551	$^2H_{11/2}$,$^4S_{3/2}$→$^4I_{15/2}$	0.0008/K (327 K)	30
Tm^{3+},Yb^{3+}	Y_2O_3 nanodisk	976	476,488	$^1G_{4(a)}$,$^1G_{4(b)}$→$^3H_6$	0.0035/K (303 K)	31
Er^{3+},Yb^{3+}	Y_2O_3 nanodisk	980	522,563	$^2H_{11/2}$,$^4S_{3/2}$→$^4I_{15/2}$	0.005/K (563 K)	32
Er^{3+},Tm^{3+}	Y_2O_3 microtube	980	524+537,552	$^2H_{11/2}$,$^4S_{3/2}$→$^4I_{15/2}$	0.0056/K (504 K)	This work
Er^{3+},Tm^{3+}	Y_2O_3 microtube	980	660,680	$^4F_{9/2(1)}$,$^4F_{9/2(2)}$→$^4I_{15/2}$	0.0282/K (22 K)	This work
Er^{3+},Ho^{3+}	Y_2O_3 microtube	980	524+537,552	$^2H_{11/2}$,$^4S_{3/2}$→$^4I_{15/2}$	0.0057/K (457 K)	This work
Er^{3+},Ho^{3+}	Y_2O_3 microtube	980	660,680	$^4F_{9/2(1)}$,$^4F_{9/2(2)}$→$^4I_{15/2}$	0.0529/K (24 K)	This work

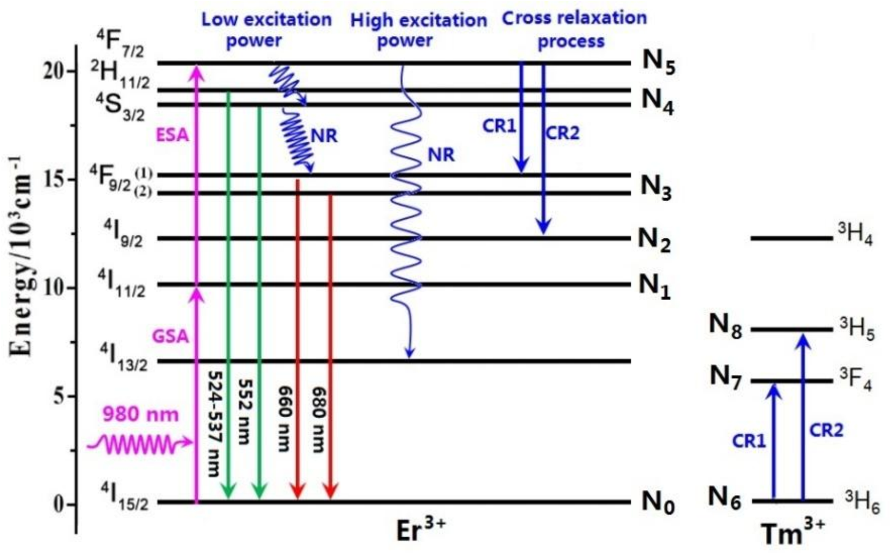

Figure S1. Energy diagram presenting energy transfer processes between Er^{3+} and Tm^{3+} at low and high excitation powers.

The theoretical model is built in Fig.S1 to have a deep understanding of the energy transfer process in Er^{3+}-Tm^{3+} system. We supposed $^2H_{11/2}/^4S_{3/2}$ or $^4F_{9/2(1)}/^4F_{9/2(2)}$ energy levels as a same level in the case of the fixed temperature. The CR effect between Er^{3+} is neglected. The corresponding rate equations are as follows:

$$\frac{dN_1}{dt} = \sigma_1 \rho N_0 - \sigma_2 \rho N_1 - A_{10} N_1 + W_{21} N_2 \tag{S1}$$

$$\frac{dN_2}{dt} = W_{c2} N_5 N_6 + W_{32} N_3 - A_{20} N_2 - W_{21} N_2 \tag{S2}$$

$$\frac{dN_3}{dt} = W_{c1} N_5 N_6 + W_{43} N_4 - W_{32} N_3 - A_{30} N_3 \tag{S3}$$

$$\frac{dN_4}{dt} = W_{54} N_5 - W_{43} N_4 - A_{40} N_4 \tag{S4}$$

$$\frac{dN_5}{dt} = \sigma_2 \rho N_1 - W_{54} N_5 - A_{50} N_5 - W_{c1} W_5 N_6 - W_{c2} W_5 N_6 \tag{S5}$$

$$\frac{dN_7}{dt} = W_{c1} N_5 N_6 + W_{87} N_8 - A_{76} N_7 \tag{S6}$$

$$\frac{dN_8}{dt} = W_{c2} N_5 N_6 - A_{86} N_8 + W_{87} N_8 \tag{S7}$$

where σ_1 and σ_2 are the light absorption cross-section of the $^4I_{15/2}$ and $^4I_{11/2}$ states, ρ is the incident pumping power density, N_0, N_1, N_2, N_3, N_4, N_5, N_6, N_7, and N_8 are the population densities of the $^4I_{15/2}$, $^4I_{11/2}$, $^4I_{9/2}$, $^4F_{9/2(1)}/^4F_{9/2(2)}$, $^2H_{11/2}/^4S_{3/2}$, $^4F_{7/2}$ levels of Er^{3+}, and $^3H_6$, $^3F_4$, $^3H_5$ levels of Tm^{3+}, respectively. W_{c1} and W_{c2} correspond to the cross relaxation rates of CR1 and CR2, respectively. The terms of W_{ij} represent the nonradiative decay rates between the levels i and j, A_{ij} is the radiative transition rates between the levels i and j.

By solving the above equations, we have

$$N_3 \approx \frac{W_{54} \rho \sigma_1 N_0 W_{43} + (A_{40} + W_{43}) W_{c1} N_6 \rho \sigma_1 N_0}{(W_{32} + A_{30})(A_{40} + W_{43})(W_{54} + A_{50} + W_{c1} N_6 + W_{c2} N_6)} \tag{S8}$$

$$N_4 \approx \frac{W_{54}\sigma_2\rho\sigma_1 N_0}{\sigma_2(A_{40}W_{43})(W_{54}+A_{50}+W_{c1}N_6+W_{c2}N_6)} \tag{S9}$$

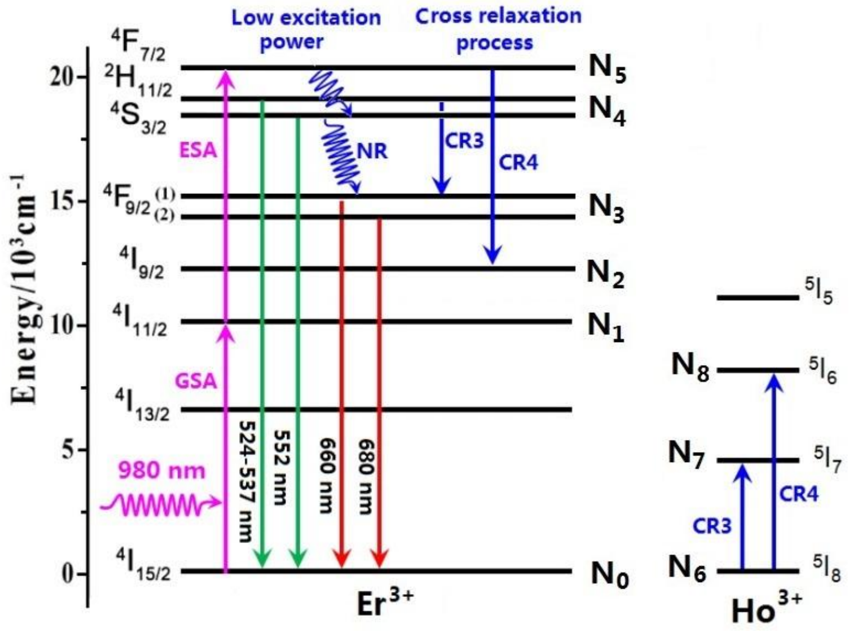

Figure S2. Energy diagram presenting energy transfer processes between Er^{3+} and Ho^{3+} at low and high excitation powers.

The theoretical model is built in Fig.S2 to understand the energy transfer process in Er^{3+}-Ho^{3+} system. The $^2H_{11/2}$/$^4S_{3/2}$ or $^4F_{9/2(1)}$/$^4F_{9/2(2)}$ energy levels are supposed as a same level in the case of the fixed temperature. The CR effect between Er^{3+} is neglected. The corresponding rate equations are as follows:

$$\frac{dN_1}{dt} = \sigma_1\rho N_0 - \sigma_2\rho N_1 - A_{10}N_1 + W_{21}N_2 \tag{S10}$$

$$\frac{dN_2}{dt} = W_{c4}N_5N_6 + W_{32}N_3 - A_{20}N_2 - W_{21}N_2 \tag{S11}$$

$$\frac{dN_3}{dt} = W_{c3}N_4N_6 + W_{43}N_4 - W_{32}N_3 - A_{30}N_3 \tag{S12}$$

$$\frac{dN_4}{dt} = W_{54}N_5 - W_{43}N_4 - A_{40}N_4 - W_{c3}N_4N_6 \tag{S13}$$

$$\frac{dN_5}{dt} = \sigma_2\rho N_1 - W_{54}N_5 - A_{50}N_5 - W_{c4}N_5N_6 \tag{S14}$$

$$\frac{dN_7}{dt} = W_{c3}N_5N_6 + W_{87}N_8 - A_{76}N_7 \tag{S15}$$

$$\frac{dN_8}{dt} = W_{c4}N_5N_6 - A_{86}N_8 - W_{87}N_8 \tag{S16}$$

Where σ_1 and σ_2 are the cross-section of the ground state absorption of $^4I_{15/2}$ and $^4I_{11/2}$, ρ is the incident pumping power density, N_0, N_1, N_2, N_3, N_4, N_5, N_6, N_7, and N_8 are the population densities of the $^4I_{15/2}$, $^4I_{11/2}$, $^4I_{9/2}$, $^4F_{9/2(1)}$/$^4F_{9/2(2)}$, $^2H_{11/2}$/$^4S_{3/2}$, $^4F_{7/2}$ levels of Er^{3+}, and $^5I_8$, $^5I_7$, $^5I_6$ levels of Ho^{3+}, respectively. W_{c3} and W_{c4} correspond to the cross relaxation rates of CR3 and CR4,

respectively. The terms of W_{ij} represent the nonradiative decay rates between the levels i and j, A_{ij} is the radiative transition rates between the levels i and j.

By solving the above equations, we have

$$N_3 \approx \frac{W_{54}\rho\sigma_1 N_0 (W_{43} + W_{c3} N_6)}{(W_{32} + A_{30})(A_{40} + W_{43} + W_{c3} N_6)(W_{54} + A_{50} + W_{c4} N_6)} \quad \text{(S17)}$$

$$N_4 \approx \frac{W_{54}\rho\sigma_1 N_0}{(A_{40} + W_{43} + W_{c3} N_6)(W_{54} + A_{50} + W_{c4} N_6)} \quad \text{(S18)}$$
